# Supplementary material for: Stacking Model‐Based Classifiers for Dealing With Multiple Sets of Noisy Labels
Source: Biom J. 2025 Mar 12;67(2):e70042. doi: 10.1002/bimj.70042 (PMC11898607; doi:10.1002/bimj.70042)
Supplement: Supplementary file 1 — Supporting Information [file BIMJ-67-e70042-s001.zip › density-based-ensemble-model-main/competitors_real_data/netlab-master/netlab-master/help/hintmat.htm]

Netlab Reference Manual hintmat


# hintmat

## Purpose

Evaluates the coordinates of the patches for a Hinton diagram.

## Synopsis

```
[xvals, yvals, color] = hintmat(w)
```

## Description

```
[xvals, yvals, color] = hintmat(w)
```

takes a matrix `w` and
returns coordinates `xvals, yvals` for the patches comrising the
Hinton diagram, together with a vector `color` labelling the color
(black or white) of the corresponding elements according to their
sign.

## See Also

`hinton`

---

**Pages:**
Index


---

Copyright (c) Ian T Nabney (1996-9)
